# Cannabinoid hyperemesis syndrome: a case report and literature review

**DOI:** 10.1192/j.eurpsy.2024.844

**Published:** 2024-08-27

**Authors:** P. Veloso, M. Gomes, R. Lopes de Dios, F. Pereira

**Affiliations:** Psychiatry, Hospital de Braga, Braga, Portugal

## Abstract

**Introduction:**

Cannabis is the most used recreational drug worldwide. Cannabinoids have long been known for their anti-emetic properties. Paradoxically, chronic cannabis consumption has been linked to inducing refractory nausea and vomiting, a condition called cannabinoid hyperemesis syndrome (CHS). CHS remains inadequately acknowledged by clinicians.

**Objectives:**

Report a CHS case and discuss this syndrome’s diagnosis, pathophysiology, and management.

**Methods:**

Collection of clinical information and review of the literature.

**Results:**

We share the case of a 38-year-old male who repeatedly recured to the emergency department (ED) due to persistent vomiting, nausea, and abdominal pain. The patient had experienced similar intermittent episodes over the past 12 years. Interestingly, the use of hot showers provided symptomatic relief. Urine drug tests consistently showed positive results for cannabinoids. During acute phases, he required supportive treatment involving fluid therapy. Long-term treatment included cannabis abstinence. CHS is defined by episodic vomiting, following prolonged excessive cannabis consumption, which is alleviated by sustained cessation of cannabis. During the acute phase of the condition, patients often find relief using hot baths and showers, which is a common behavior observed. CHS-related complications encompass acute kidney injury and severe electrolyte disturbances. CHS can result in multiple ED visits, frequent hospitalizations, extensive diagnostic evaluations, and elevated healthcare expenditures. Although the exact pathophysiology of CHS remains unclear, some mechanisms have been proposed. These include reduced gastric motility by gastrointestinal cannabinoid receptors 1 (CB1) overriding, cannabinoid lipid buildup, endocannabinoid system dysregulation, dysregulated stress response, changes in thermoregulation, modifications in the transient receptor potential vanilloid system and genetic polymorphisms in the P450 system. In the acute phase, the foremost concern is providing supportive care including intravenous hydration and electrolyte corrections. The most effective treatment for CHS is cannabis cessation. Nevertheless, there are alternative treatments that have shown promise in alleviating symptoms, such as hot water hydrotherapy, topical capsaicin, haloperidol, benzodiazepines, propranolol and aprepitant.

**Conclusions:**

As cannabis usage becomes increasingly prevalent, it becomes imperative for healthcare providers to acknowledge the long-term effects of cannabinoids, specifically regarding CHS. This diagnosis should be contemplated when evaluating patients who experience recurrent and incoercible vomiting coupled with a history of cannabis consumption. The compulsion to take hot baths or showers can serve as a noteworthy indicator for diagnosing CHS.

**Disclosure of Interest:**

None Declared

